# The contribution of *GPR98* and *DFNB31* genes to a Spanish Usher syndrome type 2 cohort

**Published:** 2013-02-13

**Authors:** Gema García-García, Thomas Besnard, David Baux, Christel Vaché, Elena Aller, Sue Malcolm, Mireille Claustres, Jose M. Millan, Anne-Françoise Roux

**Affiliations:** 1 INSERM, U827, Montpellier, F-34000, France; 2 Grupo de Investigación en Enfermedades Neurosensoriales, Instituto de Investigación Sanitaria IIS-La Fe and Centro de Investigación Biomédica en Red de Enfermedades Raras, Valencia, Spain; 3 Centre Hospitalier Universitaire, Montpellier, Laboratoire de Génétique Moléculaire, Montpellier, F-34000, France; 4 Université Montpellier 1, UFR de Médecine, Montpellier, France; 5 Clinical and Molecular Genetics, Institute of Child Health, University College London, London, United Kingdom

## Abstract

**Background:**

Usher syndrome type 2 (USH2) is an autosomal recessive disease characterized by moderate to severe hearing loss and retinitis pigmentosa. To date, three disease-causing genes have been identified, *USH2A*, *GPR98*, and *DFNB31*, of which *USH2A* is clearly the major contributor. The aim of this work was to determine the contribution of *GPR98* and *DFNB31* genes in a Spanish cohort of *USH2A* negative patients using exhaustive molecular analysis, including sequencing, dosage, and splicing analysis.

**Methods:**

Linkage analysis was performed to prioritize the gene to study, followed by sequencing of exons and intron-exon boundaries of the selected gene, *GPR98* (90 exons) or *DFNB31* (12 exons). Functional splicing analyses and comparative genomic hybridization array to detect large rearrangements were performed when appropriate.

**Results:**

We confirmed that mutations in *GPR98* contribute a significant but minor role to Usher syndrome type 2. In a group of patients referred for molecular diagnosis, 43 had been found to be positive for *USH2A* mutations, the remaining 19 without *USH2A* alterations were screened, and seven different mutations were identified in the *GPR98* gene in seven patients (five in the homozygous state), of which six were novel. All detected mutations result in a truncated protein; deleterious missense mutations were not found. No pathological mutations were identified in the *DFNB31* gene.

**Conclusions:**

In Spain, *USH2A* and *GPR98* are responsible for 95.8% and 5.2% of USH2 mutated cases, respectively. *DFNB31* plays a minor role in the Spanish population. There was a group of patients in whom no mutation was found. These findings confirm the importance of including at least *GPR98* analysis for comprehensive USH2 molecular diagnosis.

## Background

Usher syndrome (USH, OMIM 276900, OMIM 276905, OMIM 605472) is a recessive inherited disease characterized by sensorineural hearing loss (HL), visual loss due to retinitis pigmentosa (RP), and, in some cases, vestibular dysfunction. The syndrome is the most common cause of combined visual and hearing loss, accounting for more than 50% of adult cases with deaf-blindness [1]. Prevalence estimates have ranged from 3.2 to 6.2/100,000 with a recent study indicating that USH prevalence could be much higher at up to 1/6,000 [2].

Patients with USH are classified into three clinical subtypes (USH1, USH2, or USH3), based on the severity and progression of hearing impairment and presence or absence of vestibular dysfunction [3,4]. USH2, the subject of this study, is the most common type and is characterized by moderate to severe congenital HL and normal vestibular function [5]. Usually RP develops during the second decade.

Three USH2 genes are known, *USH2A*, *GPR98* (also known as *VLGR1*), and *DFNB31*. The long isoforms of *USH2A* (*USH2Ab*) and *GPR98 (VLGR1b)* encode two transmembrane proteins, usherin and G protein-coupled receptor 98, respectively, that contain large extracellular domains. *DFNB31* encodes the post synaptic density protein (PSD95), Drosophila disc large tumor suppressor (Dlg1), and zonula occludens-1 protein (ZO-1) (PDZ) domain-containing scaffold protein, whirlin. These three USH2 proteins are part of the Usher protein complex, in which USH1 and USH2 proteins are assembled in a multiprotein scaffold with a major function in the cochlea hair cells as well as in the photoreceptor cells [6-8].

Among the three known genes responsible for USH2, results from large European cohorts [9-12] have shown that *USH2A* is by far the most frequently involved gene and accounts for at least 75% of USH2 cases. Molecular analyses of the *GPR98* and *DFNB31* genes remained scarce until recently because of their minor involvement and, logistically, because of the high number of exons (n=90) to screen in *GPR98*. The most thorough study of *GPR98* and *DFNB31* was performed by Besnard et al., who reported 17 mutations in *GPR98* and two in *DFNB31* equivalent to involvement in USH2 of 6.4% and 1.3% for *GPR98* and *DFNB31*, respectively [12]. Two other analyses found a contribution of 6% or 19% for *GPR98* and 0% or 9.5% for *DFNB31*, respectively [11,13].

Recently, another gene, *PDZD7*, was shown to contribute to USH2 as a modifier of the retinal phenotype on a *USH2A* background or in digenic inheritance with *GPR98* [14]. We have previously studied the *USH2A* gene in a Spanish cohort, which accounts for 76.1% of the patients with USH2 [10], leaving a significant percentage of unresolved cases. We present in this work findings of the exhaustive mutational screening of *GPR98* and *DFNB31* performed in this *USH2A* negative cohort.

## Methods

### Subjects

Informed consent, approved by the Ethic Committee of the Hospital La Fe, was obtained for all patients and this study followed the tenets of the Declaration of Helsinki. The patients were recruited from the Federación de Afectados de Retinosis Pigmentaria de España (FARPE) and also from the Ophthalmology and ENT Services of several Spanish Hospitals as part of a large-scale study on the genetics of Usher syndrome in the Spanish population. The 19 *USH2A* negative patients genotyped in this work were previously studied in [10] and were divided as follows: 12 patients were classified as having USH2, five displayed atypical Usher syndrome, and in two cases, detailed clinical data could not be obtained. The subjects had been classified based on their clinical history and ophthalmologic, audiometric, and vestibular tests.

### Molecular analyses

Haplotypes and sequencing analyses of *GPR98* and *DFNB31* were performed as already described [12]. The conditions and the list of the primers for PCR sequencing of the two genes *GPR98* and *DFNB31* and the microsatellites used for haplotypes analyses are given in Besnard et al. [12]. Nomenclature of the variants follows the Human Genome Variation Society (HGVS) recommendations. A laboratory-designed comparative genomic hybridization (CGH) microarray chip (12×135 k), which includes all Usher genes and their 5′ and 3′ regions, was used to detect large genomic rearrangements [15].

### In silico analyses

The potential effects on splicing of any sequence variation were analyzed with the Human Splicing Finder (HSF) tool. The multistep analysis described by Baux et al. [16], and Roux et al. [15], was used to classify the variants. In particular, USMA was used to predict the impact of the missense variants on the protein structure.

### Pathogenicity grades

The classification system for unknown variants is the same as that used in USHbases and is as follows: UV1: variant certainly neutral; UV2: variant likely neutral; UV3: variant likely pathogenic; UV4: variant certainly pathogenic. This classification is in line with the guidelines published by the clinical and molecular genetics society (Best-Practice-Guidelines).

Briefly, variants were classified based on the following criteria: previously published, allele frequencies, whether they are in cis or trans to deleterious mutations/UVs, predictions from bioinformatics regarding whether the change is in a conserved region, and whether it is likely to alter the protein structure. The last two criteria are considered the main criteria.

### Minigene construction and expression

In vitro analyses were performed to evaluate the functional consequence at the RNA level of variant c.14368C>T. We used a minigene construct based on the expression vector pSPL3 [17], generated by Besnard et al. [12], which included the wild-type exon 70 and surrounding sequences of *GPR98.* The c.14368C>T variant was generated by site-directed mutagenesis (QuikChange II; Stratagene, La Jolla, CA). The minigene construct was transiently transfected into ARPE-19 cells (ATCC, CRL-2502TM) during 24 h. Briefly, 70-80% confluence cells plated in six well plates were transfected with the FuGENE6 Transfection Reagent (Roche Diagnosis, Indianapolis,IN) according to the manufacturer’s instructions. Reverse transcriptase reactions were carried out with the Superscript II Reverse Transcriptase (Invitrogen, Cergy-Pontoise, France) on total RNA extracted from cells with the Nucleospin RNAII kit (Macherey-Nagel, Hoerdt, France). Polymerase chain reactions were performed using vector-specific primers (5’-CAT CCT GGT CAG CTG GAC G-3’; 5’-GTA GGT CAG GGT GGT CAC GA-3’) and amplification products were analysed as previously described [18].

### GenBank numbers

GenBank reference sequences: *GPR98*: NM_32119.3; *DFNB31*: NM_015404.2. The +1 position corresponds to A in the ATG translation initial codon.

## Results

### Haplotype analyses

Haplotype analyses were performed as the first step at the USH2C (*GPR98*) and USH2D (*DFNB31*) loci because consanguinity was documented in some families (n=3) or because several sibs were available. Homozygosity was revealed at the USH2C locus in five families (RP1188, RP153, RP1157, RP952, RP1068, Table 1). Subsequent sequencing of the *GPR98* gene identified a homozygous mutation in all cases (see below). Haplotype analyses excluded USH2C and USH2D loci in one family, RP98. This family did not undergo subsequent investigation in this study.

**Table 1:** Genotype of the patients bearing mutations in *GPR98.*

Patient	Mutations	Diagnosis	Year of birth	Reported consanguinity
RP1068	c.17368_17369delinsTTAT /c.17368_17369delinsTTAT	USH2	1966	Yes
RP1157	c.18261delA /	USH2	1953	Yes
	c.18261delA			
RP1188	c.17204+4_17204+7del /	USH2	-	No
	c.17204+4_17204+7del			
RP153	c.6932_6939dup /	USH2	1958	No
	c.6932_6939dup			
RP952	c.12528–1G>T /	USH2		Yes
	c.12528–1G>T			
RP1590	c.10301delT /	USH	-	-
	c.12528–1G>T			
RP1634	c.17386C>T / -	USH	-	-

All listed mutations are predicted to lead to a truncated protein due to the apparition of a PTC either directly, or because of a frameshift.

### Mutational analysis

#### GPR98

Sequencing of the 90 coding exons of *GPR98* revealed in seven of the 18 patients (who had not been excluded by haplotyping for the USH2C locus) seven different mutations, of which six were novel (Table 2). Five patients were homozygotes, one patient was a compound heterozygote, and one patient carried only one identified mutation (RP1634; Table 1). Five of the patients with *GPR98* mutations were diagnosed with Usher syndrome type 2, and two patients could not be classified because of lack of clinical data (Table 1).

**Table 2:** Pathogenic mutations identified in *GPR98*

Exon	cDNA	Protein	Splice effect predicted	Reference
31	c.6932_6939dup	p.(Glu2314fs)	No	Novel
49	c.10301delT	p.(Leu3434fs)	No	Novel
IVS61	c.12528–1G>T	p.(?)	Yes	Novel
IVS79	c.17204+4_17204+7del	p.(?)	Yes	[12]
80	c.17368_17369delinsTTAT	p.(Ser5790fs)	No	Novel
80	c.17386C>T	p.(Gln5796*)	No	Novel
86	c.18261delA	p.(Gln6088fs)	No	Novel

Five of the seven mutations predict a premature termination codon, leading to a truncated protein. These variants include a small duplication, two small deletions, a deletion/insertion, and a nonsense mutation (Table 2). All were classified as a priori deleterious.

The other two pathological mutations affect splice sites, altering the correct splicing mechanisms, and were classified as UV4 (unknown variant certainly pathologic). The variant c.12528–1G>T (intron 61) was detected in two families: in a homozygous state in RP952 and in trans to c.10301delT in RP1590 (Table 3). The second splicing variant detected in a homozygous state in RP1068 is a deletion of four nucleotides (c.17204+4_17204+7del) previously described by Besnard et al. [12] that abolishes the +4/+5 positions of the 5′ splice site (SS), and results in the exon skipping of exon 79.

**Table 3:** Clinical description of the patients bearing mutations in *GPR98.*

Patient	Sensorineural hearing loss	Vestibular function	Onset of night blindness	Onset of visual field loss	Visual field	Visual acuity	Eye fundus	ERG	Cataracts
RP1068	moderate since 7 years	Normal	30	-	Marked Concentric Loss	0.25/0.25 (at 34 years)	1	-	BE
RP1157	moderate-severe	?	11	-	Marked Concentric Loss	0.16/0.16 (2010)	1	Abolish (37 years)	BE
RP1188	moderate since 1 year	Normal	17	16		0.5/1 (2002)	1	-	No
RP153	moderate-severe	Normal	16	-	Marked Concentric Loss	-	1	-	BE
RP952	moderate since 4 years	Normal	25	35	Moderate Concentric Loss (at 45 years)	0.7/0.7 (at 45 years)	1	Abolish	BE

Onset of night blindness and visual field loss are expressed in years. Eye fundus 1: Bone spicule deposits, attenuation of vessels, and pale optic nerve. ERG: Electroretinography. BE: Both Eyes. No clinical description is available for patients RP1590 and RP1634.

Seventy-five non-deleterious variants recorded in USHBases by our group or others were detected. Nineteen additional variants were identified (Appendix 1), eight of them absent from any of the databases (the Single Nucleotide Polymorphism database, 1000 Genomes, Exome Variant Server). All were classified as neutral, UV1, or UV2 based on allele frequency, bioinformatic predictions, or, in the case of c.14368C>T, in vitro experiments.

In silico analysis of the c.14368C>T variant predicted an increase in the strength of a cryptic donor splice site recognition (score of 52.41 to 79.25 for HSF and −6.61 to 1.13 for Maximum Entropy software [MaxEnt]). An in vitro splicing assay was performed to test for a splicing alteration. No altered splicing was detected. Results clearly show that c.14368C>T, identified in a single patient (RP1059), did not alter proper splicing of exon 70 in vitro.

Analysis of the 90 *GPR98* exons was completed with the CGH-array analysis for the three patients carrying a single deleterious or newly identified missense variant: patient RP1634 heterozygous for the pathological mutation c.17386C>T (p.Gln5796*; Table 1) and patients RP1059 and RP1611, heterozygous for the missense alterations c.14368C>T and c.8585A>G, respectively (Appendix 1). Deletions or duplications were not detected in any of these patients, supporting the non-pathogenicity of the two missense variants, which remained UV1 or UV2.

**Figure 1:**
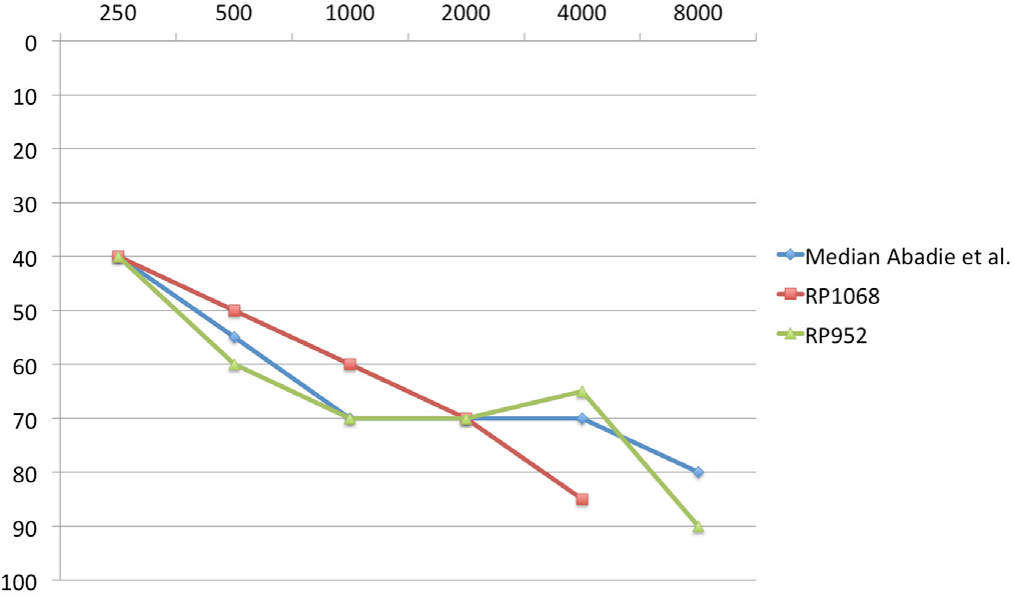
Audiograms from two patients mutated in *GPR98.* Age and sex corrected audiograms (ISO 7029) of RP952 and RP1068 mutated in *GPR98* are represented along with the median audiogram of *GPR98*-mutated patients from the Abadie et al. study [5]. Hearing loss (0–100) is in dB, and frequencies (500–8000) are in Hz.

Audiograms for two of the genotyped patients are shown in Figure 1. They are characterized by moderate to severe hearing loss with a down-sloping configuration. This is similar to that observed by Abadie et al. for patients with *GPR98* mutations [5]. In both patients, tone loss was slightly stronger at high frequencies, confirming the tendency for *GPR98*-mutated patients to present with a more severe hearing loss than those mutated in *USH2A* [5].

#### DFNB31

Mutational analysis of the *DFNB31* gene was performed in 13 patients for whom no homozygosity was detected at either locus. Fourteen different variants were identified. All but one were previously recorded in the public databases with frequencies suggestive of a benign interpretation. A single novel isocoding variant c.2112G>T, localized in exon 8, was identified in family RP1600, but a deleterious effect on splicing was not predicted with in silico analyses, and no other clear mutation was detected in this patient. This variant was not reported in the 1000 Genomes Project or in the Exome Variant Server but was considered non-pathogenic.

## Discussion

Seven deleterious mutations were identified in *GPR98.* They include small insertions and deletions, point mutations, and splicing alterations; all predicted premature termination codons. Six are new. This study raises the total number of established pathogenic mutations to 40. It confirms that the mutational spectrum of *GPR98* differs from that of *USH2A* in that no missense causative mutations were identified here. Mutations were spread throughout the whole gene, mainly localized in the terminal end [12].

In silico analyses of the new potential splice site mutation c.12528–1G>T predict that this mutation abolishes the wild-type 3′ SS of exon 62 (reducing the scores from 79.61 to 50.67 and 7.31 to −1.27, for HSF and MaxEnt matrices, respectively). The expected effects could be either a skip of exon 62 or the use of a cryptic site localized 11 nucleotides downstream from the wild-type acceptor site (increased strength from 79.26 to 82.42 and −2.6 to 2.8, for HSF and MaxEnt, respectively). In both hypotheses, this variation results in the disruption of the coding phase (deletion of 139 or 11 nucleotides) and therefore should be clearly considered pathogenic.

We observed in this cohort a high number of homozygous cases: five of the seven patients were positive for *GPR98,* as expected for rare mutations. Only three of these were reported to be consanguineous, which confirms the helpfulness of carrying out preliminary haplotype analysis.

Our overall results are very similar to those obtained in the United Kingdom [11] and in France [12]. In more than 100 patients with USH2 studied in each study, both groups found an involvement of about 80% for *USH2A* and 6% for *GPR98* with mutations in *DFNB31* being absent or negligible. In a much smaller sample (21), Bonnet et al. reported a lower contribution of *USH2A* (57%) with four patients and two patients for *GPR98* and *DFNB31*, respectively [13].

Recently, Vaché et al. described the first example in Usher syndrome of a deep intronic mutation causing activation of a pseudoexon, through analyses of RNA from nasal cells in a patient with only one mutation detected in the *USH2A* gene [19]. The same type of mutations could arise in *GPR98,* as patient RP1634 carries a single mutation, the absence of additional genomic rearrangements has been tested, and no mutation was identified in *PDZD7* (not shown). Interestingly, several patients with a single *GPR98* mutation have been identified in other studies [11,12]. Limitations of molecular studies to the sequencing of all the coding exons and their boundaries together with the CGH array leave some unresolved cases that require further studies at the RNA level. A mutation in the 5′ or 3′ untranslated region cannot be excluded.

Twelve USH2 patients remained with no mutation in either of the USH2 genes. These patients will undergo next generation sequencing (NGS) applied to “Usher-exome” (i.e., targeted exome of the Usher genes) as this approach is becoming available. Several patients have been reported who have presented with a clinical subtype of Usher in which the mutated gene is usually responsible for a different subtype. Several examples have been identified [11,13,20,21]. Although they remain rare, they represent a real pitfall in terms of molecular diagnosis using conventional approaches such as Sanger sequencing focusing on cascade sequencing of the different genes.

## Appendix 1.

Novel variants detected in *GPR98* classified as non-pathologic. To access the data, click or select the words “Appendix 1.”
